# Microplastics in freshly fallen snow: How may it adversely impact human health and exacerbate the COVID-19 crisis?

**DOI:** 10.1016/j.amsu.2022.104276

**Published:** 2022-07-31

**Authors:** Ayesha Liaquat, Aleena Kashif, Sushma Rathi, Alishba Raza

**Affiliations:** a Dow Medical College, Dow University of Health Sciences, Baba-e-Urdu Road, Saddar, Karachi, 74200, Pakistan; b Medical College, Aga Khan University, Karachi, 74800, Pakistan

The use and production of plastics have increased exponentially in the past few decades owing to a surge in human population and industrialization. Consequently, over the last twenty years, plastic waste has also doubled in quantity, of which only 9% is being recycled [1]. However, it is the spike in one particular type of plastic, microplastics (MPs), which has alarm bells ringing throughout the globe. In a recent study by the University of Canterbury, 29 particles of MPs were found per liter of freshly fallen snow in Antarctica-one of the most remote areas in the world [2]. This discovery is a clear indicator of an alarmingly high level of airborne MPs in our surrounding.

MPs are man-made, microscopic plastics that are <5 mm in size [3] and have been found in water bodies, rocks, and living organisms [4]. While the adverse effects of MPs on human health have not been as extensively researched and documented as their impact on the environment, the risk they pose is far too significant to ignore [5]. Human beings are constantly exposed to MPs: inhalation of airborne MPs from city dust, synthetic fibers or rubber tires [4]; ingestion of contaminated food and water [5]; and skin contact with nanoplastics and microbeads, two types of microplastics found in toiletries [6]. Studies show that nanoplastics can form complexes with blood proteins such as albumin and globulin which could result in vascular occlusion [7]. Moreover, in various *in vitro* investigations, MPs have induced inflammation and oxidative stress leading to cell poisoning [6]. Additionally, people who work in textile or plastic industries have occupational exposure to MPs and may exhibit signs of respiratory infections, lesions [8], and even complete respiratory failure. The latter was the cause of two fatalities following prolonged exposure to polyacrylate nanoparticles in an air spray unit lacking proper ventilation [4]. Furthermore, particle pollution due to vehicular traffic is linked with an increased predisposition to neurodegenerative disorders such as Alzheimer's disease and dementia. MPs are speculated to have the same effect on the nervous system as demonstrated by an *in vivo* study where they adversely impacted neuronal activity [8]. The severity of all the aforementioned health impacts relies heavily on the extent of exposure to MPs and individual susceptibility [8].

Additionally, the role of MPs as vectors in disease transmission is another cause for concern. Previously, MPs have acted as vectors for pathogenic bacteria genera such as Vibrio and Pseudomonas [9]. On the surface of MPs, several pathogenic microorganisms have been observed including the family Rhodobacteraceae, the algae group Bacillariophyta, and members of the Campylobacteraceae [10]. Similarly, MPs may also transmit viruses such as SARS-CoV-2. Recent studies have shown the presence of coronavirus RNA on particulate matter in the air which also acts as a carrier for airborne MPs, thereby increasing the chances for the two to bind together. Moreover, airborne MPs shed from contaminated personal protective equipment (PPE) such as gloves can also be expected to transport the virus to distant areas through airflow [10]. Indeed, a field study conducted in France demonstrated that airborne MPs could be carried as far as 95 km away from the source through the wind [11]. Since coronavirus can survive approximately 72 h on a plastic surface (the longest survival time for the virus on any object) and only 3 h in aerosol droplets, airborne MPs carrying coronavirus could be a way to transmit the virus over a longer time and distance, thereby inflicting disease upon regions which were previously partially or completely unaffected by it [12].

Hence, steps must be taken to alleviate this looming threat to human health. Accordingly, in China, scientists are working on developing a small robot fish made of biocompatible materials that can absorb MPs in water, thereby reducing microplastic pollution in water bodies [13]. Moreover, Prata et al. identified the insufficiency of techniques to detect and identify harmful MPs in the air [4], therefore funding research to improve these techniques can ensure that global efforts to reduce MPs are precise and effective. Identification of these MPs can help inform the replacement of major sources, for example, replacing synthetic textiles with organic materials that shed biodegradable microfibers instead. Furthermore, sustainable fashion choices may be made globally to reduce the number of new clothing items which release more microfibers than fabrics that have been washed a few times [14]. In the UK, the fitting of microplastic filters in washing machines has been suggested to reduce the amount of MPs polluting water [15]. Since 34.8% of microplastic waste is estimated to come from fabrics [16], implementing these suggestions will significantly reduce the overall load of primary MPs on the environment. Microbeads, another significant source of microplastics found in toiletries and makeup products, have been banned in Canada, the UK, Ireland and the USA [17]. Single-use plastics which produce secondary MPs have also been banned in several countries including the UK, European Union, Canada, and Costa Rica. Since these bans have successfully reduced the production of MPs [17], they should be placed on a global scale to eliminate these large contributors of microplastic waste. Also, adaptation strategies can be globally implemented to help reduce the impact of airborne MPs on the respiratory health of humans. The high indoor concentrations of MPs [4] indicate the need for proper ventilation measures for indoor spaces as well as textile and plastics factories which present an occupational hazard to workers. This is because airborne microplastic concentration is inversely proportional to wind speed [4]. Lastly, proper disposal of PPE must be ensured to prevent the potential spread of COVID-19.

To sum up, the surge in microplastic concentration in the environment, as well as its potential adverse effects on human health, should not only be considered a matter of concern but effective mitigation strategies must also be deployed immediately before the matter gets out of hand.

## Ethical approval

This paper did not involve patients, therefore no ethical approval was required.

## Sources of funding

No funding was acquired for this paper.

## Author contribution

**Ayesha Liaquat:** conception of the study, major drafting of the work, literature search, final approval and agreeing to the accuracy of the work. **Aleena Kashif:** conception of the study, literature search, major drafting of the work, final approval and agreeing to the accuracy of the work. **Sushma Rathi:** conception of the study, major drafting of the work, final approval and agreeing to the accuracy of the work. **Alishba Raza:** conception of the study, drafting of the work, final approval and agreeing to the accuracy of the work.

## Trial registry number

1. Name of the registry:
2. Unique Identifying number or registration ID:
3. Hyperlink to your specific registration (must be publicly accessible and will be checked):

## Guarantor

Ayesha Liaquat, Aleena Kashif, Sushma Rathi, Alishba Raza.

## Consent

This study was not done on patients or volunteers, therefore no written consent was required.

## Declaration of competing interest

The authors declare that there is no conflict of interest.
